# The Embodied-Enactive-Interactive Brain: Bridging Neuroscience and Creative Arts Therapies

**DOI:** 10.3389/fpsyg.2021.634079

**Published:** 2021-04-29

**Authors:** Sharon Vaisvaser

**Affiliations:** School of Society and the Arts, Ono Academic College, Kiryat Ono, Israel

**Keywords:** brain function, integration, embodiment, predictive processing, simulation, brain-to-brain coupling, creative arts therapies

## Abstract

The recognition and incorporation of evidence-based neuroscientific concepts into creative arts therapeutic knowledge and practice seem valuable and advantageous for the purpose of integration and professional development. Moreover, exhilarating insights from the field of neuroscience coincide with the nature, conceptualization, goals, and methods of Creative Arts Therapies (CATs), enabling comprehensive understandings of the clinical landscape, from a translational perspective. This paper contextualizes and discusses dynamic brain functions that have been suggested to lie at the heart of intra- and inter-personal processes. Touching upon fundamental aspects of the self and self-other interaction, the state-of-the-art neuroscientific-informed views will shed light on mechanisms of the embodied, predictive and relational brain. The conceptual analysis introduces and interweaves the following contemporary perspectives of brain function: firstly, the grounding of mental activity in the lived, bodily experience will be delineated; secondly, the enactive account of internal models, or generative predictive representations, shaped by experience, will be defined and extensively deliberated; and thirdly, the interpersonal simulation and synchronization mechanisms that support empathy and mentalization will be thoroughly considered. Throughout the paper, the cross-talks between the brain and the body, within the brain through functionally connected neural networks and in the context of agent-environment dynamics, will be addressed. These communicative patterns will be elaborated on to unfold psychophysiological linkage, as well as psychopathological shifts, concluding with the neuroplastic change associated with the formulation of CATs. The manuscript suggests an integrative view of the brain-body-mind in contexts relevant to the therapeutic potential of the expressive creative arts and the main avenues by which neuroscience may ground, enlighten and enrich the clinical psychotherapeutic practice.

## Introduction

The integration of knowledge from recent neuroscience research into Creative Arts Therapies (CATs) practice and education, in a multidisciplinary translational approach, can be transformative for advancing the comprehension and treatment of different mental states and disorders. CATs disciplines, such as dance/movement therapy, art therapy, music therapy, drama therapy, and psychodrama, seek to engage clients holistically across somatic, cognitive, emotional, cultural, aesthetic, and social aspects of the self (Dunphy et al., 2019). CATs include verbal and non-verbal forms of expression and communication, accentuated embodied knowledge and memory, multisensory engagement, presence and liveness, and the transcendent qualities of imagination and creativity (Malchiodi, 2005; Van Lith, 2015; Czamanski-Cohen and Weihs, 2016; Gerber et al., 2018; Samaritter, 2018). The comprehensive essence of the psychotherapeutic treatment could interweave with neuroscience that addresses the mechanistic understanding of brain functions. A deeper understanding of the neurobiological underpinnings of the subjective and intersubjective experiences, as well as their maladaptive shifts, may have great benefits and help improve the therapist’s observations, goals and interventions, to optimally help clients. Moreover, the knowledge generated from neuroscientific studies anchors the appreciation of ways in which the therapeutic work might influence brain function and the validity of our profession and thus advances professional identity (King et al., 2019).

Indeed, the assimilation of neuroscientific knowledge into psychotherapeutic practice has received growing interest over the past decade, both in general (Javanbakht and Alberini, 2019) and specifically with relation to the different CATs modalities (Homann, 2010; Belkofer and Nolan, 2016; Berrol, 2016; O’Kelly et al., 2016; Payne, 2017; King et al., 2019). The current manuscript aims to describe and discuss main characteristics of brain function and their applicability to the therapeutic work, addressing intertwining themes, presented in the following sections. The first section elaborates on the neuroscientific meaning of embodiment; the second introduces and deliberates on the predictive nature of the mind and the formation and reformation of internal models; the third articulates and discusses predictive processing mechanisms in the context of the psychic apparatus; the fourth explores developmental and therapeutic implications of the brain’s predictive mechanisms; and the fifth section focuses on the relational account of neural functioning and the underpinnings of empathy. The fundamental brain mechanisms are conceptualized and tailored to the understanding of their clinical relevance, ultimately acknowledging the neuroplastic potential of CATs.

## The Embodied Brain and the Grounding of the Mind

Advancements in the field of neuroscience lend substantial support to the embodied nature of brain mechanisms (Fabiani, 2015; Kiverstein and Miller, 2015). This implies that neural processing, bodily action, and environmental forces are constantly and complexly combined in constant dynamic feedback loops. The brain does not merely passively perceives input and controls body action, but rather actively creates perceptual experiences from sensory stimuli accumulating from the body’s internal milieu (interoceptive and proprioceptive) and from the surroundings (exteroceptive), in a generative manner. Mental representations dynamically evolve over time, in the form of nerve impulses that propagate in circuits and functional network assemblies, in order to anticipate, decode and respond to complex concrete (physical) and abstract (social) variables in the environment, based on prior information (Ju and Bassett, 2020; Teufel and Fletcher, 2020). Relevant to this argument, it has been acknowledged that in natural settings, brain activity is neither predefined nor fixed, but rather continuous and transient (Dmochowski et al., 2012; Zioga et al., 2018). The reciprocity between the human mind and the sensing active body, within the relational environment, underscores the need for holistic and integrative mental health approaches, such as CATs.

From the earliest foundation of the sense of self, neural processes related to multisensory perception give rise to the embodied, spatially located, self-conscious experience (Park and Blanke, 2019). Accordingly, the qualia of the mental experience of feeling were shown to be associated with the unique topography of the concurrent bodily sensations (Nummenmaa and Saarimäki, 2019). Subjective feelings were suggested to be elicited by the perception of emotion-related bodily states that reflect changes in the skeletomuscular, neuroendocrine (hormonal), and autonomic peripheral nervous systems (Levenson, 2003; Barrett et al., 2007; Damasio and Carvalho, 2013). Moreover, neural structures responsible for sensory, motor and emotional experiences are also involved in attributing linguistic meaning, expressing those experiences in words (Buccino et al., 2016). Respectively, language processing re-enacts sensorimotor, emotional, and introspective experiences (Pulvermüller and Fadiga, 2010). Indeed, sensory perception, movement, emotion, and cognition are interdependent processes that dynamically influence each other and are supported by cohesive brain networks (Pessoa, 2013). Well beyond the direct anatomical pathways that signals to and from the body, emotion is closely linked to transient bodily states and actions through functional interactions between the regions implicated in emotion and those that are chiefly important for mental operations, traditionally labeled as “cognitive,” such as decision making, spontaneous thought or mind-wandering (Babo-Rebelo et al., 2016; Pessoa and McMenamin, 2017). Correspondingly, there is a paradigm shift toward an action-oriented view of cognition (Engel et al., 2016). The well-supported concept of “grounded cognition” stresses that cognitive processes lie upon meaningful interactions with a dynamic environment and cannot be reduced to thinking-related mental representations (Engel et al., 2013). There is a close and causal relationship between sensorimotor and conceptual systems of the brain (Buccino et al., 2016). The brain possesses an ability to take our physical experiences and use it metaphorically for abstract thinking as basis of imagination and creativity (Wang et al., 2019).

This canonical framework implies that it is within the embodied actuality, through the subjective, lived, bodily experience, that we may induce awareness and change the way we perceive, think and feel about ourselves and others. This coincides with and highlights the uniqueness of the arts and the therapeutic potential of the embodied enactive CATs (Koch and Fuchs, 2011; Koch, 2017). Within the context of a containing relationship, CATs offer clients the opportunity to explore, articulate and express experiential content, grounded in the body and connected with representations of feelings and mental perceptions of the self and the outer world.

The following sections will develop these ideas through an intriguing neuro-functional account that is ultimately becoming a general and unifying explanation of brain function, and, by extension, dysfunction, with emerging clinical applications.

## The Predictive Brain and Experience-Based Generative Representations

An increasingly supported hypothesis in contemporary neuroscience, which concurs with the conceptualization of the “embodied brain,” converges on the idea that the brain runs internal models that function as probabilistic (Bayesian) inference for incoming sensory information from inside and outside the body (e.g., Friston, 2010; Clark, 2013). For the purpose of maintaining homeostasis and minimizing “free energy,” the brain makes meaning of the current situation, by re-implementing past experiences, and proactively tailoring the body’s responses accordingly (i.e., enacts allostasis, Barrett, 2017b). These past experiences are embodied representations that continuously anticipate upcoming sensory events and the best action to deal with those events, i.e., “predictive processing.”

As aforementioned, connectedness, at a structural and functional level, is a fundamental aspect of brain architecture. Neural activity cascades through hierarchical pathways, from lower level sensory and motor processing toward a functional spectrum of progressively higher level abstract representations. Accordingly, the constructive, non-deterministic nature of sensory perception emerges from continuous functional interaction between higher and lower levels of the neural processing hierarchy. The predictions are originated in cortical regions that unconsciously generate, in “top-down” (descending) pathways, expectations (beliefs) of hidden causes of the unfolding sensory events. The perception of actual sensory input, passed on in a “bottom up” (ascending) pathways, to the brain is then constantly shaped and modified by descending prior expectations. “Bottom-up” sensations are matched with “top-down” predictions, creating an interplay between forward and backward flow of information (Friston, 2005, 2010; Barrett and Simmons, 2015). Anticipated inputs confirm the predictions and become meaningful sensations, while unanticipated information or discrepancy between what was expected and what occurred becomes a prediction error – a “surprise.” The mismatch, in turn, serves as a driver for change, in order to minimize free energy, by actively interacting with the environment to better match predictions (“active inference”), or by updating the relevant predictions to accommodate unexpected signals, an opportunity to induce remodeling, adjusting and modifying the internal models (i.e., predictions) of the embodied exchange with the world (Clark, 2013).

Notably, predictive processing connects to embodied and enactive approaches, it is not merely a view of the brain as reducing the uncertainty of its sensory observations but rather concerns the active, selective sampling of the world by an embodied agent (Ramstead et al., 2020). In other words, the brain is no longer viewed as a passive system that generates complex representations over time, with top-down processes playing a modulatory role on the accumulating bottom-up stimulation, but rather an active organ that is constantly predicting its future state and stimulation. Actions, and their accompanying mental events, begin as top-down representations in the brain, constructed from past experiences that are tested against the state of the (social) world. The dependence on embodied priors occurs implicitly, without the requirement to explicitly remember past events (Hutchinson and Barrett, 2019). The comparison of predictions with incoming sensory information forms prediction errors, which serve as corrective feedback and can be minimized using two strategies: (1) changing sensory input through action or (2) changing the internal models of the world (prediction signals) (Venter, 2021).

The ontology of the human mind, through the lens of “predictive processing,” provides a powerful framework that captures fundamental aspects of psychic functioning and yields considerable ground to experientially oriented integrative therapeutic approaches, such as CATs. These brain mechanisms and brain-body-mind intersections are explored in more detail below.

## The Multifaceted Predictive Brain in the Psychic Landscape

The predictive function of brain, or the way the brain actively handles uncertainty in dynamic relationships with the body and the environment, is a multifaceted, multidisciplinary, and multilevel account of the mind. It may map proto- and core aspects of the self, reflected in neural processes, which may further explain why predictive coding and error processing have been suggested to lie at the heart of a wide range of mental health conditions and disorders (Harrison et al., 2019; Smith et al., 2021), and how it applies to our therapeutic work.

Multisensory integration underpins minimal (pre-reflective) experiential aspects of the bodily self, including senses of agency and ownership, based on a model that successfully predicts the sensory consequences of one’s own movement, intentions in action, and sensory input (Hohwy, 2007; Limanowski and Blankenburg, 2013). This also establishes forms of self-awareness, enabling the recognition of the self, as the most accurate explanation of the inferred and modeled hidden cause of one’s sensory experience (Apps and Tsakiris, 2014). It explains how we make sense of the world, already observed in infant learning, navigating the dynamic physical and social environment (Köster et al., 2020). The predictive work in progress, thus, applies not only for maintaining life and for learning how to meet organismic needs in the world, but also for selfhood (self-organizing existence within a world that can be separated from the self) that conforms to goal-directed notions of intentionality (Solms and Friston, 2018).

Emotion has been eagerly advocated to belong at the very heart of the predictive-coding embodied nexus (Friston et al., 2018; Miller and Clark, 2018). According to these view of emotion, on-going changes in the peripheral body actively contribute to the creation of emotional experiences, in a constructive way, using accumulated knowledge from prior experiences (Barrett, 2017b).

Internal models of the body in the world (i.e., predictions) are generated, for example, in cortical regions of the brain that constitute the salience network, which is involved in affective experience, including the anterior insula and anterior cingulate cortex. These regions are continually informed by bodily changes and mediate interoception, supporting awareness and response to relevant internal or external stimuli, imbuing these stimuli with emotional weight, and using this information to guide attention and behavior (Barrett et al., 2016; Seeley, 2019).

The dynamics of predictive processing relate also to self-referential processing and the way we integrate memory representations with ongoing events and envision our future. In this account, top-down associative predictions also involve the regions of the medial prefrontal cortex: the ventromedial prefrontal cortex, which creates Gestalt representation of how an organism is situated in its environment and the subjective value of environmental stimuli, which then drives predictions about future events (Roy et al., 2012; Dohmatob et al., 2020); and the dorsomedial prefrontal cortex, which supports higher level evaluative control and may subserve the representation and assessment of self and other’s action considerations (Alexander and Brown, 2015). Both of these medial regions of the prefrontal cortex (i.e., the ventral and dorsal midline regions), along with the posterior cingulate cortex, are also part of the default mode network (DMN), a unique brain network that performs baseline mental activities, supporting free-ranging thought, spontaneous introspection, autobiographical memory, episodic future thinking, and mentalizing (Andrews-Hanna et al., 2014; Raichle, 2015; Wen et al., 2020).

In the process of generating predictions and at moments of uncertainty and surprise (i.e., prediction-errors), these aforementioned cortical brain regions communicate with sensorimotor cortices and with sub-cortical regions, such as the hippocampus, supporting associative memory; the amygdala, a key region of emotional processing; and the ventral striatum, integrating affective and rewarding/motivational information. These functional communications likely involve the temporal integration of incoming information with internal representations stored in memory, recently shown in naturalistic ongoing events, suggested to support narrative integration (Brandman et al., 2021). Moreover, an emotional experience manifests when there is resonance across hierarchical levels, that is, conceptualization and abstraction supported by higher-level DMN function make concrete, present moment, multisensory lower-level features meaningful as discrete emotions, in a given context (Satpute and Lindquist, 2019).

Both emotional awareness and emotion differentiation, also termed emotional granularity, defined by the ability to accurately distinguish between specific emotions like anger, sadness or frustration, rely on this internal model of the body in the world created by the brain (Barrett, 2017a). Correspondingly, alexithymic features, including affective agnosia, an impairment in knowing how one feels, have been linked with this neuroscientific theory of how “predictive processes” create emotional experiences (Duquette, 2020). Accordingly, poor emotional awareness and differentiation are considered transdiagnostic features of mental disorders and may act as either a vulnerability or maintenance factor (Kashdan et al., 2015; Lane et al., 2015b; Smith et al., 2018). Understanding such mechanisms could represent an important step in identifying which mechanisms are operative in different individuals and how they might be targeted on an individual basis within therapy (Smith et al., 2021). Poor emotional awareness may relate, for example, to overly precise prior expectations for somatic threats, leading to somatization (high anxiety sensitivity), or to highly imprecise predictions and emotion-concept acquisition due to consistently high levels of threat or environmental unpredictability (Smith et al., 2021).

These neural mechanisms linked to psychological constructs further lead to implications regarding the underlying developmental trajectories and the therapeutic work, elaborated on in the next section.

## The Development and Use of a Predictive Brain in the Therapeutic Realm

Before delving into the development of predictive abilities and its manifestation in the relational context, let us recognize again that the brain’s *modus operandi* is situated, constantly receiving information from, responding to, updating expectations and monitoring actions in accordance with the ongoing communicative signals (Hari et al., 2015). What we perceive of the world in any given moment, then, depends, critically, on the nature of our prior learning about the world and object relations. In order to increase predictability, we explore the environment (beginning with the infant exploring the mother’s breast) and the mind of others with our unconscious phantasies and proto-representations, building a repertoire of “priors” to make future inferences (Holmes and Nolte, 2019). Predictable input from the caregiver enables the brain to begin differentiating “self” versus “non-self” causes of sensations, securing an experience of ownership and agency (Fonagy et al., 2002; Seth, 2013; Di Plinio et al., 2020). The probabilistic predictions we generate may be experienced as daydreams or fantasies (Bucci and Grasso, 2017), this coincides with the aforementioned involvement of the default mode network.

With early life experience, the brain assembles predictive models, which underpin the development of social concepts and skills (Atzil et al., 2018), linked to the way we mentalize about own and others’ internal bodily and mental states (Koster-Hale and Saxe, 2013; Fotopoulou and Tsakiris, 2017; FeldmanHall and Shenhav, 2019). Primary embodied interactions permit the “mentalization” of bodily signals across exteroceptive and interoceptive sensory modalities, transforming rough perceptions into subjective feelings (Fotopoulou and Tsakiris, 2017). Correspondingly, the process was recently metaphorically linked to Bion’s postulation of the way “alpha function” (i.e., maternal “reverie” generating top-down predictions) processes the infant’s projected “beta fragments” (unnamed “bottom-up” raw sensory impressions) (Bion, 1962; Holmes and Nolte, 2019; McVey et al., 2020). Both the “alpha function” and “predictive processing” framework focus on the linking of implicit expectations and the actual realization, based on prior learning and inference, to produce the meaning-saturated elements of thought. The importance of such linking relies on the flexible and mutual flow of information between predictions and experience, rather than being frozen in maladaptive patterns (as in depression) or disrupted and fragmented (as in psychosis) (McVey et al., 2020).

Developmental studies emphasize the role of attachment figures, with whom one could appropriately place epistemic trust (Fonagy and Allison, 2014), in the progressive refinement of the internal self-model via predictive processing mechanisms, in the course of psychological development (Palmer et al., 2015; Fotopoulou and Tsakiris, 2017; Pereira et al., 2019). In accordance, in cases of insecure attachment, the possibilities for active (or enactive) inference are compromised due to the absence of a secure base for exploration. The limited extent and range of sensory sampling of the environment, confines the variety of “priors” available to account for them (Holmes and Nolte, 2019). The therapeutic secure relationship might alter these dynamics of brain function drawing on neuroplasticity (Pascual-Leone et al., 2005).

By enabling a safe and trustful environment and by “loaning” the therapist’s brain functions, creating a “reverie” that can offer therapists a means to enter the predictive moment, psychotherapy mobilizes the active inference in the context of intimate relationships (Holmes and Nolte, 2019; McVey et al., 2020). Moments of creative not-knowing may emerge and hence the need for active exploration, innovation and generative possibilities. In that sense, CATs may encompass both strategies to engage predictive processing neurodynamics – sampling new sensory input through action (active inference) and shaping the internal models of the world (prediction signals) through meaning-making (Venter, 2021).

The experimental aspects of CATs provide the opportunity for sensorimotor exploratory actions, expanding the individual’s repertoire of experiences in a secure, accepting, and facilitating relationship. Thus, the neuroplastic therapeutic processes enables both the “breaking” (i.e., creative destruction) of priors and the “making” (i.e., creative construction) of new ones, seeking for the optimal individually tailored balance between predictability (a therapeutic setting that does not overwhelm the client) and opportunities for prediction error- uncertainty, surprise and play.

Given that emotions, as predictions, are embodied conceptual categories built with experiential multisensory features, preparing the body for action while simultaneously making meaning of the incoming sensory array (Hoemann et al., 2020), valuable opportunities for emotional processing in CATs may arise. The usage of art forms as a vehicle for affective expression may enhance emotional awareness and acceptance. Attention to bodily sensations or unconscious somatically experienced knowledge, can further be transferred to explicit thought through language and symbolic formations, interweaving affective, cognitive and social domains (Czamanski-Cohen and Weihs, 2016; Koch, 2017). The process can also update resistant prior beliefs and in doing so, alternative expectations about the subjective experience can be generated.

Moreover, the predictive processing account of emotion might interlace with theories of aesthetic emotions, such as the feeling of “being moved,” savored for their own sake, linked with subjectively felt intensity, or rewarding emotional arousal (Menninghaus et al., 2019; Zickfeld et al., 2019). Aesthetic experiences, engaging the brain’s reward network, are laden with tension, fluctuations in uncertainty, generating prediction errors (“surprises”) that create a yearning for resolution, triggering predictive processes directed at future events of emotional significance (Koelsch, 2014; Lehne and Koelsch, 2015; Shany et al., 2019). Minimization of prediction error involves the embodied capacity to generate experience through action, thereby fostering creativity (Daikoku, 2019; Schiavio and Benedek, 2020). In this sense, CATs may tap into the intrinsic motivation to seek predictive progress and offer clients opportunities to venture out of predictable zones (Gottlieb et al., 2013). Successful predictions remain implicit, it is prediction errors that attract consciousness (Solms, 2015). The discovery of affective dynamics, unfolding through prediction errors, including negatively valenced contents, validates self-existence and the cognitive-affective schemata with which one experiences and navigates the world (Van de Cruys et al., 2017). Through embodied aesthetic expression and impression in CATs, that involve symbolizing, meaning-creating processes (Koch, 2017), predictive progress is being made, enlivening curiosity, creativity and sense of mastery.

Such therapeutic processes may also drive associative plasticity to update internally generated predictions that draw on memory traces (Barron et al., 2020). Indeed, an error in prediction, between expected and current events, affects different stages of learning and memory (Dudai, 2012) and can also drive the updating of consolidated memories in the process of reconsolidation, when memory is labile and susceptible, during its reactivation (Sevenster et al., 2014; Exton-McGuinness et al., 2015; Fernández et al., 2016; Gershman et al., 2017; Lee et al., 2017). In this regard, CATs may assist the formulation of a reconsolidated narrative, while processing traumatic memories also in the implicit realm, using the art forms that incorporate body movement (Hass-Cohen, 2016; Gerge et al., 2019; Hass-Cohen and Findlay, 2019; Perryman et al., 2019). Notably, the process of reconsolidation depends on emotional reactivation, without necessarily explicitly recalling the event, as well as on the recognition of a mismatch or disconfirming information (prediction error). Further activation of new emotional experiences allows the situation to be experienced and understood in a different way, adding safe elements to a threatening memory, working through the emotional consequences of the new learning in a variety of contexts and focusing on creating a more coherent narrative (Lane et al., 2015a; Lane, 2018; Hass-Cohen and Findlay, 2019).

These dynamic processes emerge within the context of a therapeutic relationship, moving us toward second-person neuroscience perspective, learned through real-time social encounters (Schilbach et al., 2013; Redcay and Schilbach, 2019). Focusing on interactive phenomena occurring in the relational matrix, the next section explores the neural mechanisms underscoring the capacity to grasp the mental states of others, i.e., mirror neuron and mentalizing systems; as well as the intriguing interbrain coupling through shared experiences.

## The Relational Brain and the Link Between Subjectivity and Intersubjectivity

Intrapsychic experiences are interwoven with and dependent on relational and intersubjective contexts. These may be played out in the therapeutic relationship and transference-countertransference interaction, supported by the therapist’s presence and empathic attunement. Social neuroscience offers a window into the origins and dynamics of this relational matrix.

The conceptualization of the brain as embodied and the agent-environment dynamics of the predictive processing account of brain function, relates also to its social essence, being interconnected, interactive and intercommunicative. Humans are social species and the complex specialization for social stimuli processing encompasses regulation from the neurotransmitter to the neural network level, resulting in a “social brain” (Dunbar, 2009). Consequently, deficits in these processes may result and relate to diverse neuropsychiatric disorders (Porcelli et al., 2019). Indeed, robust literature suggests that human brains are wired to connect, dependent on ongoing social transaction and thereby should be considered in the context of other human brains. It has become increasingly clear that the brain is practically sensitive to social presence, whereby the mere presence of another person can alter brain activity (Verbeke et al., 2014). Furthermore, it is the non-verbal aspects of social interaction and embodied relational experiences that lay the foundation for the development of brain function (Hari et al., 2015; Atzil et al., 2018).

This interpersonal resonance is anchored by brain mechanisms of simulation- the vicarious representations of the bodily and emotional states of others through mirror neuron systems, and synchronization- the inter-individual neuronal coupling of brain activity during interaction.

Mental simulation theories of action understanding, based on mirror mechanisms of the brain, suggest that observed actions are matched onto the observer’s motor system (in mirror neurons in the premotor and parietal cortex), thereby allowing an understanding of the intentions behind these actions (Gallese et al., 1996; Rizzolatti et al., 1996; Rizzolatti and Sinigaglia, 2016). Furthermore, research has demonstrated that the human brain is also endowed with mirror mechanisms in the domain of emotions. Simulation describes the mapping of not only movements, but also sensations and emotions of others, mapped onto the observer’s visceral and somatosensory systems (Gallese and Sinigaglia, 2011). This implies that overlapping nervous structures involved in the subjective experience of emotions are also active when such emotions are recognized in others (Gallese, 2003; Keysers and Gazzola, 2009). This process, termed embodied simulation, recruits sensory-motor regions (e.g., the premotor cortex) along with visceromotor brain regions (e.g., the anterior insula) mediating interoception. Accordingly, neuroimaging studies have shown an overlap between these brain regions implicated in self and bodily experiences and those underpinning mental inferences about the affective states of others (Decety, 2015; Abraham et al., 2019); a shared neural representations for self- and empathetic emotions, including pain, and positive or negative emotions (Zaki et al., 2016).

Embodied simulation, therefore, recreates the other’s emotional state in one’s brain, a mechanism that enables individuals to resonate with other’s mental state and emotions, and grounds experience in the present moment (Gallese, 2014). These simulations of self and other, as ongoing, intrinsic activity, function as internal models (i.e., predictions) that construct emotions and perceptions, and guides actions (Chanes and Barrett, 2016). The simulation process in the parental brain, for example, was shown to be critical for grounding a “shared space” in the brain that underpins the capacity to build and maintain attachment (Feldman, 2017). Accordingly, studies found that this embodied simulation network of a parent also supports the child’s long-term sociality, well-being, and health, with participation of the neurotransmitter and hormone oxytocin, which underpins the development of trust and bonding (Abraham et al., 2019). Neural mechanisms that underlie simulation may explain individual variability in social functioning in healthy, at-risk and clinical populations (Masson et al., 2019; Lincoln et al., 2020).

The simulation mechanisms signify intercorporeality – the mutual resonance of intentionally meaningful sensorimotor behaviors – as a primordial source of intersubjectivity (Gallese, 2014). This concurs with the methods of CATs, demonstrating how deeply our making sense of others’ living and acting bodies is rooted in the power of re-using our own motor, emotional and somatosensory resources (Gallese, 2013). Notably, this mechanism plays a key role in our aesthetic experience, in the engagement of bodily memories and in imaginative associations (Gallese, 2017).

Empathic concern requires motivational investment and thereby is also supported by brain regions associated with reward processing (Singer and Klimecki, 2014; Weisz and Zaki, 2018). Furthermore, studies suggest a division between regions responding preferentially to internal states of the others’ bodily grounded emotional experience, versus internal states of others’ thoughts (Lombardo et al., 2010; Bruneau et al., 2012; Kanske et al., 2015; Spunt et al., 2016). Indeed, in addition to embodied emotional resonance, empathic attunement entrails cognitive understanding, which involves the activation of a theory of mind (ToM) or “mentalizing” network in the brain. This “mentalizing” network mainly includes the temporoparietal junction (TPJ), and regions of the aforementioned default mode network (DMN, mainly concerns midline anterior and posterior cortices), involved in the attribution of mental states, beliefs and intentions to others. The DMN is associated with distinguishing internal from external information, as well as generating and contemplating on thoughts and intentions of both the self and significant others (Alcalá-López et al., 2019; Dohmatob et al., 2020; Wen et al., 2020).

Importantly, mentalization during empathic engagement refers also to the attribution of emotions, wishes, desires, and needs (Fonagy et al., 2002) and involves automatic, embodied aspects, as well as more controlled reflective aspects (Luyten and Fonagy, 2015). The neuroimaging literature may help us understand this phenomenon better, suggesting that distinct neural networks are involved in self-knowing and knowing others, differentiating between affective simulation processes, i.e., shared representations for firsthand and vicarious experiences of affective states, and cognitive mental state attribution, which relies more on symbolic and abstract processing (Shamay-Tsoory, 2011; Raz et al., 2014; Luyten and Fonagy, 2015). As aforementioned, these networks show overlapping activations for mentalizing about the self and others (Lombardo et al., 2010). Accordingly, both the sense of self and the sense of others rely on the functional integration and segregation of default mode, sensorimotor, affect-related (insula, cingulate cortex) and executive brain networks (Di Plinio et al., 2020). This also concurs with the idea that the capacity for mentalizing is acquired in the context of attachment relationships (Fonagy and Luyten, 2016), since, as depicted above, attachment is founded on the caregiver’s capacity for empathic engagement (supported by the simulation and mentalizing networks) (Abraham et al., 2018), establishing the mechanism that creates our internal models (i.e., predictions).

It is not surprising then that the DMN, a core brain system for processing information about the self and about others, has emerged as a key system underlying multiple psychopathologies, mainly due to abnormal connectivity and decreased segregation between the DMN and other functional brain networks (Xia et al., 2018). This was shown, for example, in autism (Padmanabhan et al., 2017; Reiter et al., 2019), Schizophrenia (Kottaram et al., 2019), depression (Scalabrini et al., 2020; Zhou et al., 2020) and PTSD (Akiki et al., 2018).

Acknowledging the interplay between the aforementioned affective and cognitive dimensions of empathy, emphasized through the lens of brain systems, is particularly valuable in the context of the therapeutic relationship. Such dynamics may reflect the oscillation and tension between subjective and objective positions through the course of psychotherapy, and phenomena such as identification and transference-countertransference (including somatic) transactions. Within this interchange, a key issue is that the therapist processes her/his own implicit (body-based) and background (conscious but in the attentional background) feelings to make sense of the current interactive experience (Lane, 2018). Distinguished from emotional contagion, for other-directed empathetic concern to arise, along with the grounding of empathy in our own emotional experiences through shared representations, there needs to be a clear self-other distinction, or the capacity to correctly distinguish between our own affective representations and those related to the other (Lamm et al., 2016). This is presumably accomplished by inter-network connectivity and functional integration among brain systems, such as the embodied simulation (salience) and mentalizing networks (Levy et al., 2019).

Intriguingly, during real-time moments of affect sharing, a fascinating phenomenon of neurophysiological synchrony between people, analogous to a wireless communication, was operationalized and empirically observed (Anders et al., 2011; Hasson et al., 2012). This spontaneous and dynamic concordance in brain activity between individuals was found in brain regions of embodied simulation, reward/motivation and mentalization functions (Feldman, 2017), with regions of the prefrontal cortex most commonly implicated in this “brain-to-brain coupling” (Reindl et al., 2018; Azhari et al., 2020b; Behrendt et al., 2020). Notably, the coordinated or synchronized brain activation across people, i.e., intersubject correlation, was suggested as a mechanism of transmission of shared meaning and common interpretation that goes beyond cultural and linguistic boundaries (Honey et al., 2012): Moments of neural synchrony may be driven by shared attention to external stimuli in the environment or directly mediated by person-to-person communicative signals, through which interaction is conveyed (e.g., eye contact, facial expression, bodily gestures, vocal prosody). Correspondingly, the intersubjective oscillatory brain response has been related to empathic relationships and bonding (Wheatley et al., 2012; Hu et al., 2017), shown to be indicative of rapport (Ellingsen et al., 2020), emotion co-regulation and social learning, through mutual attention, and communication (Leong et al., 2017; Levy et al., 2017; Pratt et al., 2018; Reindl et al., 2018; Piazza et al., 2020). Recently, this implicit interpersonal neural resonance was suggested as the basis of attachment (Schore and Schore, 2008; Siegel, 2015; Long et al., 2020) and also affiliated with the development of resilience (Feldman, 2020). Accordingly, parenting stress was shown to have adverse effects on mother-child brain-to-brain synchrony (Azhari et al., 2019) and anxiously attached mothers exhibit less synchrony with their child, suggested to indicate less attunement to his/her mental state (Azhari et al., 2020a). Strengthened synchronization was presented when events were accompanied by strong emotions (Nummenmaa et al., 2012, 2014), social proximity (Parkinson et al., 2018), behavioral synchrony (Kinreich et al., 2017; Levy et al., 2017) and group bonding (Yang et al., 2020). Importantly, there seems to be an agreement on the fact that the brain-to-brain networks become increasingly efficient and integrated as the level of interaction between subjects intensifies (Falk and Bassett, 2017).

Within empathic relationships, moments of interpersonal match/synch are integrated with moments of mismatch, with relation to behavior, neurophysiology and mental states (Feldman, 2020). These notions provide biological and scientific evidence to Winnicott’s conceptualization on the critical continuity of being in the presence of a non-impinging good enough other as the basis for play and creativity (Winnicott, 1971). Furthermore, interbrain coupling is experience-dependent, demonstrating its specific relevance to CATs; it increases with diversity of repertoire, symbolic level and degree of mutuality, tuning the social brain (Levy and Feldman, 2019). Artistic experiences were shown to induce brain-to-brain coupling, e.g., while creating, performing or listening to music together (Babiloni et al., 2012; Müller et al., 2013; Zamm et al., 2018; Sachs et al., 2020), viewing dance performance (Jola et al., 2013; Herbec et al., 2015), in shared movement (Reddish et al., 2013) and in interacting audiences during free viewing of live cinema (Zioga et al., 2018). In the context of the therapeutic relationship, the contribution of creative processes to the intersubjective neuropsychological boding is thus compelling. Synchrony is rooted in biological rhythms grounding this experience in the physical and concrete and enabling the history of the relationship to resonate within “moments of meeting,” as “implicit relational knowing” (Stern et al., 1998; Bruschweiler-Stern et al., 2007; Feldman, 2020).

## Summary and Conclusion-Creative Arts Therapy and Neuroplasticity

Recent advances in understanding of brain mechanisms that underlie mental processes, as well as psychotherapeutic change, might have significant impact on CATs practice; not only for arriving at the most accurate and holistic account of case formulation and presentation, but also for treatment and intervention.

The invigorating present views of brain function that endorse its embodiment, dynamic processes of prediction, remodeling, resonance and interconnectedness, ground the intra- and inter- psychic features and highlight the therapeutic potential of the expressive creative arts. Moreover, these neuroscientific accounts emphasize the pre/non-verbal dynamics that play an important role in shaping self-experience and development and are an integral part of the CATs setting, were creativity, intimacy, and aliveness might emerge. The ability of the brain to plastically develop, adapt and change is a fundamental well-recognized phenomenon (Pascual-Leone et al., 2005). In accordance with the brain’s embodied engagement with the inner and outer environment, plastic processes were shown to be highly action-dependent and modifiable by experience (Fields, 2008; Tomassy et al., 2016; Sampaio-Baptista and Johansen-Berg, 2017). Moreover, neuroplastic processes are heavily influenced by environment, culture and the accumulation of experiences of production and appreciation of the arts (White-Schwoch et al., 2013; Bolwerk et al., 2014; Nadal and Chatterjee, 2019; Teixeira-Machado et al., 2019; Zamorano et al., 2019).

The exploratory, integrative nature of CATs may be linked to various dynamic brain mechanisms; enabling the brain to become more sensitive to experience-based context, broadening sensory sampling, processing of emotional salience and interoceptive awareness, promoting agency and shaping the interpersonal world to revise “priors” and reconsolidate memories in the light of experience. Therapeutic attunement encompasses means for interpersonal brain-to-brain coupling and the integration of the lived experience with reflective, meaning-making processes that might promote communication between different functional systems. These include neural systems related to the multidimensional sense of self and self-other relationship, supporting emotional awareness, motivational investment, mirroring or embodied simulation mechanisms and mentalization. Further research that evaluates the presented mechanisms will be valuable and useful to the field. Recent developments of mobile brain/body imaging technology, for example, may yield more extensive understandings of brain function in natural settings and in relational contexts, also during real-time artistic creation (King, 2018; Cruz-Garza et al., 2019; King and Parada, 2020).

Our task then, as therapists, is to tap into the brain’s potential for change and mobilize plasticity in the process of therapeutic development. Emotionally attuned experiential practice, enriched in multisensory and expressive tools, which provides a safe space for self and self-other explorations, might do just that. Neuroscience-informed therapy, acknowledging dynamic brain responses that underlie the subjective and intersubjective experiences, would enable deeper understandings of these intra- and inter-personal psychodynamics.

## Author Contributions

The author confirms being the sole contributor of this work and has approved it for publication.

## Conflict of Interest

The author declares that the research was conducted in the absence of any commercial or financial relationships that could be construed as a potential conflict of interest.
